# Molecular replacement then and now

**DOI:** 10.1107/S0907444913011426

**Published:** 2013-10-18

**Authors:** Giovanna Scapin

**Affiliations:** a Global Structural Chemistry, Merck and Co. Inc, 2000 Galloping Hill Road, Kenilworth, NJ 07033, USA

**Keywords:** molecular replacement, models, accuracy, quality

## Abstract

The ‘phase problem’ in crystallography results from the inability to directly measure the phases of individual diffracted X-ray waves. While intensities are directly measured during data collection, phases must be obtained by other means. Several phasing methods are available (MIR, SAR, MAD, SAD and MR) and they all rely on the premise that phase information can be obtained if the positions of marker atoms in the unknown crystal structure are known. This paper is dedicated to the most popular phasing method, molecular replacement (MR), and represents a personal overview of the development, use and requirements of the methodology. The first description of noncrystallographic symmetry as a tool for structure determination was explained by Rossmann and Blow [Rossmann & Blow (1962 ▶), *Acta Cryst.* **15**, 24–31]. The term ‘molecular replacement’ was introduced as the name of a book in which the early papers were collected and briefly reviewed [Rossmann (1972 ▶), *The Molecular Replacement Method.* New York: Gordon & Breach]. Several programs have evolved from the original concept to allow faster and more sophisticated searches, including six-dimensional searches and brute-force approaches. While careful selection of the resolution range for the search and the quality of the data will greatly influence the outcome, the correct choice of the search model is probably still the main criterion to guarantee success in solving a structure using MR. Two of the main parameters used to define the ‘best’ search model are sequence identity (25% or more) and structural similarity. Another parameter that may often be undervalued is the quality of the probe: there is clearly a relationship between the quality and the correctness of the chosen probe and its usefulness as a search model. Efforts should be made by all structural biologists to ensure that their deposited structures, which are potential search probes for future systems, are of the best possible quality.

## Abstract 2

A brief overview, with examples, of the evolution of molecular-replacement methods and models over the past few years is presented.

## 1. Introduction

**Figure 1:**
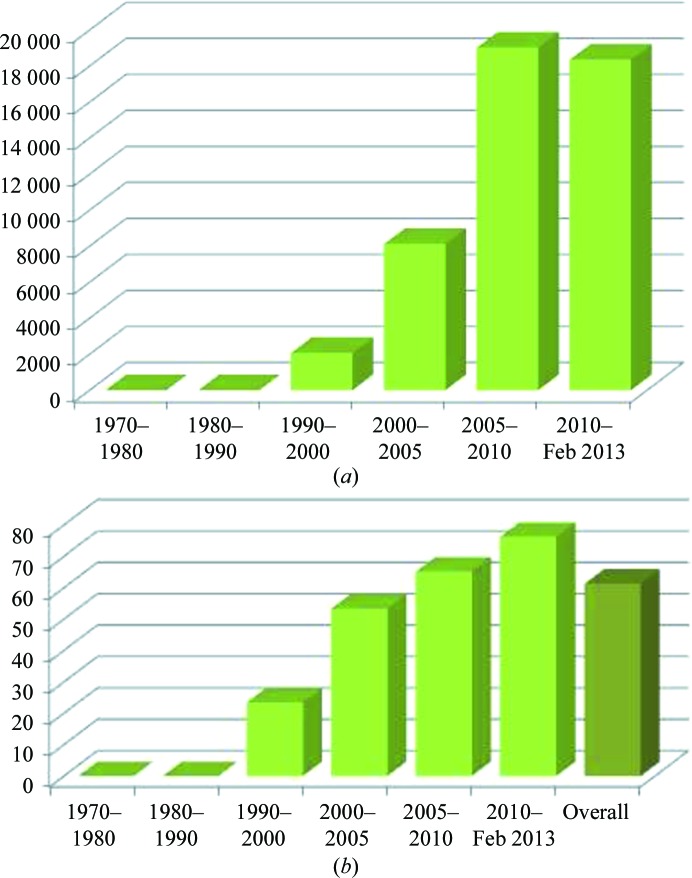
(*a*) Number of X-ray structures that have been solved using MR from the inception of the PDB (1970) until 15 February 2013: the total number approaches 78 000. (*b*) Number of X-ray entries that report ‘molecular replacement’ as the method used to solve the structure as a percentage of the total number of X-ray structures deposited in the PDB to 15 February 2013 : almost 60% of the structures have been solved using MR and in the past two years they account for greater than 70% of all depositions.

The original concept of molecular replacement (MR) was introduced in the early 1960s; the first paper mentioning a way to solve a crystal structure using a search model was likely to have been a paper by Huber (1965 ▶). The name ‘molecular replacement’ was introduced as the title of a book in which the early papers were collected and briefly reviewed (Rossmann, 1972 ▶). As subsequently stated (Rossmann, 2001 ▶), Rossmann’s original intent in generating the term molecular replacement was ‘to cover all methods that utilize NCS whether within or between crystal forms’, since the mathematical procedures required apply equally to the situation in which a known homologous structure can be used as a search model and to phase determination in the presence of noncrystallographic symmetry. Since then the term ‘molecular replacement’ has been largely limited to the case in which an unknown structure is to be solved using a known search molecule and has become the most used method for solving ‘new’ structures: not just novel structures but also structures derived from different constructs, different complexes or different crystal forms. Fig. 1 ▶(*a*) shows the distribution of protein structures (in the PDB) that have been solved using MR since the inception of the PDB (1970) until 15 February 2013. The growth is exponential, and it is even more compelling if we look at the number of structures solved using MR compared with the entire body of X-ray-derived structures (Fig. 1 ▶ *b*): overall, almost 60% of the structures have been solved using MR, and in the past two years they account for over 70% of all depositions. This impressive growth has been made possible by a dramatic evolution of both computer hardware and software, which has resulted in a faster, more flexible and in many cases fully automated methodology, and a similarly dramatic evolution and improvement in the type, quantity and quality^1^ of the starting models. Numerous other reviews of MR have been written (Rossmann, 1990 ▶, 1995 ▶). In addition, there have been two previous CCP4 Study Weekends devoted to MR (in 2001, with the proceedings reported in the October 2001 issue of *Acta Crystallographica Section D*, and in 2007, with the proceedings reported in the January 2008 issue of *Acta Crystallographica Section D*). This paper aims to be a review of the development of the method, with examples taken from the literature and the author’s own experience.

## 2. Methods

Analysis of the distribution in the PDB of the major software used to solve structures using MR from 1970 to date shows that over 90% of the structures to date have been solved using either *X-PLOR*/*CNS* (Brünger, 1992*b* ▶), *AMoRe* (Navaza, 1994 ▶), *MOLREP* (Vagin & Teplyakov, 2010 ▶) or *Phaser* (McCoy *et al.*, 2007 ▶). Although *X-PLOR*/*CNS* and *AMoRe* have been widely used in the past, *Phaser* and *MOLREP* are probably the current cutting-edge choice for routine molecular-replacement efforts and some examples of these packages are briefly described below. Before that though, it is probably worth briefly mentioning the earlier programs, which are in many ways the precursors of what we have today.

### 2.1. Initial programs

The *PROTEIN* package (Steigemann, 1974 ▶) included Lattman’s rotation and translation functions (Lattman & Love, 1970 ▶; Lattman, 1972 ▶) and could be used to solve MR problems, but lacked some of the features (consistency, speed and automated screening of a large number of results) that are desirable for a program suite. Paula Fitzgerald’s *MERLOT* (Fitzgerald, 1988 ▶) was the first program to provide most of the necessary features in one program suite, including rapid screening of a large number of potential MR solutions generated, for example, by noisy rotation-function results. *MERLOT* included two programs for calculating the rotation (including Crowther’s fast rotation function; Crowther, 1972 ▶), three methods for solving the translation problem [the Lattman translation function, a packing function (Hendrickson & Ward, 1976 ▶) and an *R*-value mapping program] and a program for optimizing a potential solution. The programs were applicable to both macromolecular and small-molecule structure determinations. Although *MERLOT* lacks automation to the same degree as found in more modern programs, it has been used well into the 21st century (for example, for PDB entry 4dne; Panwar *et al.*, 2012 ▶). If the crystal system contains more than one monomer per asymmetric unit, the use of a locked rotation function (Tong & Rossmann, 1990 ▶) or the inclusion of noncrystallographic symmetry constraints in MR searches can provide faster results. *GLRF* and *TF*, Tong’s rotation-function and translation-function programs based on this principle, are included in the two suites *Replace* (Tong, 1998 ▶) and *COMO* (Tong, 2005 ▶). A handful of programs (including *CNS* and *X-PLOR*) provide ways to perform six-dimensional searches. Among these programs it is worth mentioning *BRUTE* (Read & Schierbeek, 1988 ▶), and *EPMR* (Kissinger *et al.*, 1999 ▶). *BRUTE* carries out a brute-force rotation/translation search by computing a Patterson correlation coefficient and *EPMR* is a general-purpose molecular-replacement program that uses an evolutionary search algorithm to simultaneously optimize the orientation (rotation) and position (translation) of a search model. Other possibilities for six-dimensional searches are *BEAST* (Read, 2001 ▶), a likelihood-based molecular-replacement program that can also perform a brute-force search, *SOMoRe* (*Search and Optimization Molecular Replace­ment*; Jamrog *et al.*, 2003 ▶) and *Queen of Spades* (Glykos & Kokkinidis, 2000 ▶), which represents an attempt to write a multi-dimensional, multi-model, space-group general, molecular (re)placement program. Six-dimensional searches are usually very time-consuming because of the dimensionality of the search^2^ and this may be a reason why their use has never really taken off.

### 2.2. Molecular replacement in *X-PLOR*/*CNS*

**Figure 2:**
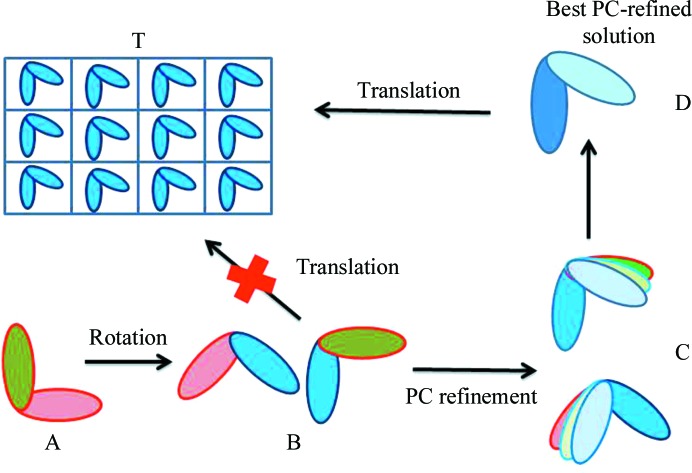
Schematic representation of the Patterson refinement procedure as applied to the MR solution of Fab domains. The search model (A) has an Fv and an Fc domain [red and green, connected by a hinge (the elbow angle)]; the relative orientation of the two domains in the search model is different from the orientation in the target (T). The rotation search performed with the intact Fab may provide several solutions, each matching one individual domain (B). These solutions are not easily distinguishable or rankable, and subsequent translation searches usually fail. PC refinement allows sampling of the relative orientation of the Fv and Fc regions, from which the ‘best model’ can be identified (D). This model can then easily generate a solution in the translation search.

*X-PLOR* (Brünger, 1992*b* ▶), which successively evolved into *CNS* (*Crystallography & NMR System*; Brünger *et al.*, 1998 ▶), is a software package for computational structural biology developed by Axel Brunger at Yale University, with specific emphasis on X-ray crystallography and nuclear magnetic resonance of biological macromolecules. The MR portion of the program uses the classical Patterson-based rotation-function (Rossmann & Blow, 1962 ▶) and translation-function (Crowther & Blow, 1967 ▶) implementations, which tend to run slowly. Its main advantage was the incorporation of Patterson correlation refinement, which allowed optimization of internal degrees of freedom in a model. The concept of Patterson-correlation (PC) refinement of a rotation-function result was first mentioned by Fujinaga and Read and was implemented in their program *BRUTE* (Fujinaga & Read, 1987 ▶). Axel Brunger suggested including ‘Patterson refinements’ of a large number of the highest peaks of a rotation function as a new search strategy to improve the search model before translation searches (Brünger, 1990 ▶). An early application of this method, and what made it well known, was the solution of structures of Fab domains (see, for example, Ban *et al.*, 1994 ▶). Fragment antigen-binding (Fab) domains are the part of an antibody that contains the sites that can bind to antigens. They are composed of one constant and one variable domain from each heavy and light chain of the antibody (Putnam *et al.*, 1979 ▶). The constant domain is usually referred to as the Fc region. The variable domain is referred to as the Fv region and is the most important region for binding to antigens. The Fv region and the Fc domain behave as ‘rigid bodies’ connected by a restricted axis of movement: the ‘hinge’ (represented in Fig. 2 ▶ as the elbow angle between the two domains). The rotation search may provide several solutions (matching each individual domain) that are not easily distinguishable or rankable, and subsequent translation searches usually fail. PC refinement allows the identification of the ‘best model’, or the best relative orientation of the variable and constant regions, which can then easily generate a solution in the translation search. This method has produced spectacular gains, and indeed for more difficult cases the unique *CNS* combination of enhanced signal-to-noise analysis of the rotation-function peaks, PC refinement and the fast translation function was a very attractive and much faster alternative when compared with full six-dimensional searches (Grosse-Kunstleve & Adams, 2001 ▶). Nevertheless, *CNS* and *X-PLOR* have very slow molecular-replacement implementations compared with programs such as *AMoRe* and *Phaser*, and this may be the reason behind their diminished usage in recent years.

### 2.3. *AMoRe*

**Figure 3:**
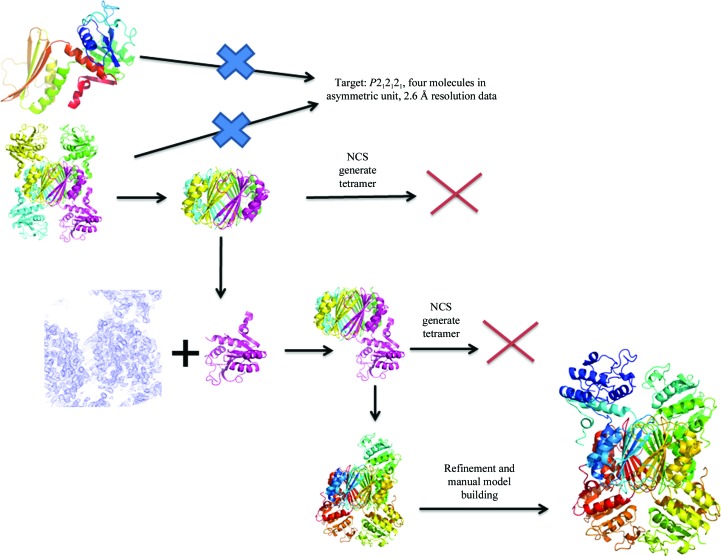
Solution of the structure of the ternary complex of *Escherichia coli* dehydrodipicolinate reductase: following the positioning of the central core, the structure was completed using a single N-terminal domain as a search model and the difference Fourier maps generated from the partial solution as the search space. NCS was then used to place two of the remaining three N-terminal domains. The fourth was built into available density following several rounds of refinement of the partial model. The final model revealed that three of the four subunits are in a closed conformation in the ternary complex, with both cofactor and substrate bound to the enzyme, while the fourth subunit is unliganded and in an open conformation.

*AMoRe* (Navaza, 1994 ▶, 2001 ▶) is a complete package that is fast and very flexible and is included in the *CCP*4 suite. It uses a modified Crowther fast rotation function (Crowther, 1972 ▶) and classic translation functions. It is very fast, and a key component of the speed is the fact that structure factors for the model are tabulated on a fine grid corresponding to a large ‘unit cell’: all subsequent structure factors required for the searches are obtained by interpolating into this table. *AMoRe* extended the *MERLOT* idea of allowing the rapid screening of a large number of potential MR solutions generated, for example, by noisy rotation-function results. In addition to the standard MR procedures, it allows the use of the locked rotation function when noncrystallographic symmetry is available, performs rigid-body refinement of the solutions and checks for symmetry clashes. It allows a search for more than one type of model to assemble multidomain solutions. It is also possible to use entities different from the standard PDB model and reflection file both as input data and search models, thus allowing greater flexibility in solving complex problems, a unique advantage in the late 1990s and early 2000s (before the advent of more sophisticated and automated suites). The following is just one example that illustrates this versatility. Scapin *et al.* (1997 ▶) reported the structure of the complex of dehydrodicolinate reductase, an enzyme involved in the *de novo* biosynthetic pathway of lysine (Scapin & Blanchard, 2006 ▶), with its cofactor NADPH and a substrate analogue. The native enzyme is a 120 000 Da molecular-weight tetramer consisting of identical subunits. Each subunit consists of two domains connected by two flexible hinge regions; in the initial binary complex structure (DHPR bound to NADPH) the tetramer is generated by crystallographic symmetry. Tetramer formation involves extensive interactions between the C-­terminal domains of the monomers (Scapin *et al.*, 1995 ▶). A partial solution for the ternary complex was obtained using the C-terminal core of the binary complex, but placement of the four N-terminal domains proved to be difficult. Finally, the structure was solved using a single N-terminal domain as a search model and the difference Fourier maps generated from the partial solution as the search space (a procedure similar to a ‘phased’ rotation and translation search). The final model revealed that in the ternary complex three of the four subunits were in a closed conformation, with both cofactor and substrate bound to the enzyme, while the fourth subunit was unliganded and in an open conformation, suggesting that the enzyme undergoes a major conformational change upon the binding of both substrates (Fig. 3 ▶).

The use of *AMoRe* peaked between 2000 and 2005 (over 41% of the structures solved using *AMoRe* were deposited in this time frame), but its use has been steadily decreasing since 2005, probably owing to the development of other suites such as *MOLREP* and *Phaser*.

### 2.4. *MOLREP* and *Phaser*

*MOLREP* and *Phaser* are probably the current cutting-edge choice for routine molecular-replacement efforts, mostly owing to their full automation and ease of use. The first paper describing *MOLREP* was published in 1997 (Vagin & Teplyakov, 1997 ▶): in the view of the author, *MOLREP* can be seen as a more sophisticated and more versatile version of *AMoRe*, of which it retains many of the functionalities, with some useful improvements. It can be run as a fully automated package, including an automatic choice of all parameters and a soft resolution cutoff (instead of the normal low-resolution cutoff, it uses a special coefficient that allows the removal of structure factors in this resolution range without introducing a series-termination effect). In addition to the standard rotation and translation searches, it allows phased rotation and translation functions, and a locked cross-rotation function that can use as input the peak-list output of the self-rotation function. The self-rotation calculation also outputs a PostScript representation of the results, which is visually extremely clear. Another interesting feature is an original full-symmetry translation function combined with a packing function. Information from the model already placed in the cell is incorporated in both the translation and the packing functions and, if the initial number of monomers is known, it is possible to locate all of the monomers in one simple run. In addition, it automatically chooses from symmetry-related models the solution closest to the molecule(s) that have already been placed, thus avoiding the need for the researcher to run symmetry-based coordinate transformation to reposition the new solution.

*Phaser* (McCoy *et al.*, 2007 ▶) is, in the view of the author, probably the most efficient molecular-replacement package available to date. It is included in both the *PHENIX* (Adams *et al.*, 2010 ▶) and the *CCP*4 software suites. It is actually a general macromolecular phasing tool, since it provides both molecular-replacement and experimental phasing methods. The phasing algorithms in *Phaser* were developed using maximum-likelihood and multivariate statistics and have proven to be significantly better than traditional methods. In the molecular-replacement mode, rotation and translation functions are followed by a packing function, which is used to identify the solutions with a minimal number of C^α^ clashes within a given distance (and thus likely to be the most correct): although the process of actually counting the clashes can be slow compared with computing a collision function, as is performed in *MOLREP*, the packing analysis provides a very powerful constraint on the translation function and in some cases provides a way to identify potentially correct solutions within a large set of incorrect solutions. When space-group ambiguity is present, *Phaser* allows all potential space groups to be scanned (or just those input by the user). *Phaser* can use single or multiple initial models (to search, for example, for hetero-assemblies).

Both *MOLREP* and *Phaser* are included in *MrBUMP* (Keegan & Winn, 2007 ▶) and *BALBES* (Long *et al.*, 2008 ▶), two automated molecular-replacement pipelines available from the *CCP*4 suite that starting from a target sequence and experimental structure factors will search for homologous structures in the PDB, create search models from the template structures, perform molecular replacement and test the solutions with several rounds of restrained refinement (Keegan *et al.*, 2011 ▶).

## 3. Search models

### 3.1. Sequence identity *versus* structure similarity

**Figure 4:**
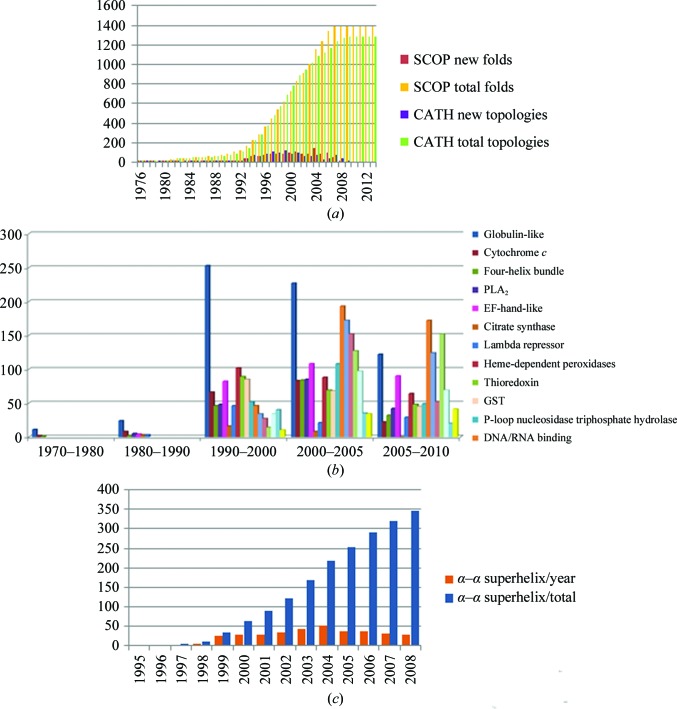
(*a*) Distribution of ‘new’ and total SCOP folds (red and yellow) and ‘new’ and total CATH topologies (purple and green) in the PDB. This graph was generated using the tools available in the ‘PDB Statistics’ page of the RSCB PDB (http://www.rcsb.org; Berman *et al.*, 2000 ▶). There has been no new fold reported since 2008 and no new topology since 2009. (*b*) Distribution of new ‘all-α’ folds over the years: the large majority were discovered between 1990 and 2000, and between then and now the distribution of folds is basically unchanged. (*c*) Yearly and total reports for the α–α superhelix fold (as defined in SCOP). Even if the total number of folds has not changed, the number of structures within the fold has increased.

It is well known that the more similar, both in primary and tertiary structure, the search model is to the target, the more likely it is that a solution to an MR problem can be found. It has generally been accepted that a 1.5 Å r.m.s.d. between the search model and the target is the lowest limit at which a related structure can be used as a search model. However, the r.m.s.d. is a *post mortem* evaluation of how similar the probe and target are, and the only initially available indicator of closeness is the sequence identity. According to the Chothia equation (Chothia & Lesk, 1986 ▶), a 1.5 Å r.m.s.d. corresponds to ∼29% sequence identity, which loosely translates into saying that a search model has to be at least 30% identical to the target to be a good search model. This is not always the case, however: a high sequence identity can still lead to a high r.m.s.d. if relative domain movements or variations in loop positions are present. On the other hand, molecules with a much lower identity can be good search models if three-dimensional similarity is retained. Fig. 4 ▶(*a*) shows the distribution of ‘new’ and total folds in the PDB both as SCOP (Murzin *et al.*, 1995 ▶) folds and CATH (Orengo *et al.*, 1997 ▶) topologies. No new folds have been reported since 2008 and no new topologies since 2009, but for MR purposes the important fact is that even if the total number of folds has not changed, the number of structures within a fold has increased. Figs. 4 ▶(*b*) and 4 ▶(*c*) show, for example, that even if the large majority of new ‘all-α’ folds were discovered between 1990 and 2000 and the distribution of folds is basically unchanged between then and now, the number of structure within each fold (the α–α superhelix fold in Fig. 4 ▶ *c* being just an example), is much higher now than just five years ago. In 2000 there were about 50 examples of the α–α superhelix fold; today there are over 350. This provides a much finer sampling of the three-dimensional space, and even if the primary-sequence identity between the target and the probe is much lower that the desired 25–35%, the chances of finding a probe with a similar three-dimensional structure are increasing. Most of the programs have ways to take this three-dimensional sampling into consideration, either by using structural alignments or multiple models or other knowledge-based modification of the search models. Various programs in *CCP*4 can be used to perform probe modification: for example, *CHAINSAW* (Stein, 2008 ▶) can be used to prune side chains based on a given alignment, *PDBCUR* provides various analyses and manipulations of PDB files, including *B*-factor analysis and ways to cut out residues/loops if their *B* factors are above an acceptable threshold, and *PDBSET* allows the removal of waters and other small molecules. The *Rosetta* suite (Das & Baker, 2008 ▶), which was initially developed for *de novo* protein structure prediction, has methods for homology modeling and protein design that can modify the starting probe and has been proven to be efficacious in solving complex molecular-replacement problems (Kaufmann *et al.*, 2010 ▶; DiMaio *et al.*, 2011 ▶). 

**Figure 5:**
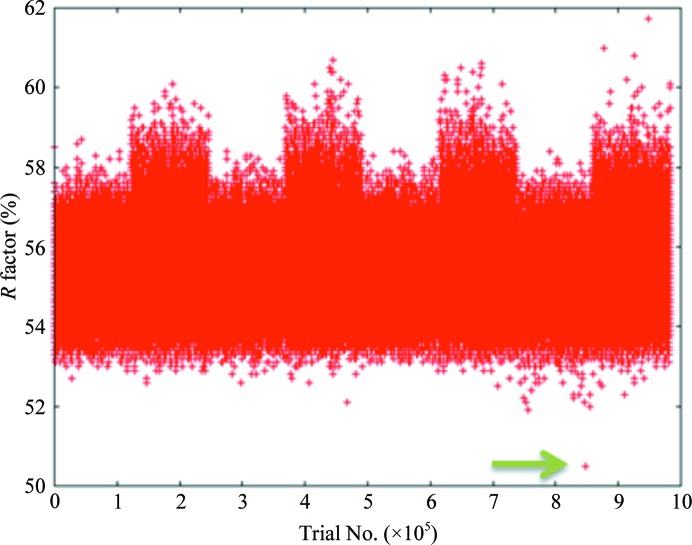
Distribution of *R* factors (as calculated by *REFMAC*) *versus* trial number for the brute-force approach molecular-replacement experiment described in the text. Only one solution clearly differentiated itself from the others (green arrow) and corresponded to a search probe with a sequence identity of less than 13% but a final r.m.s.d. on C^α^ atoms of 1.18 Å.

When everything else fails, the increase in computer power and CPU accessibility make the brute-force approach (using every possible search model available to solve a problem) more and more feasible. Such an approach was used by C. Strickland and T. Fishmann (personal communication) to solve the structure of a novel kinase. A text search of the PDB using the words ‘human protein kinase’ returns close to 3000 hits, including Ser/Thr kinases, tyrosine kinases, histidine kinases and receptor and nonreceptor kinases: although not all are unique structures, there still is a good primary and tertiary structural sampling. Initially, a standard strategy was used: using primary-sequence alignments, 29 potential search models were chosen with sequence similarity between 25 and 35%. None of them provided a good solution using standard MR programs and procedures. A fully automated search [using *AMoRe* as the MR program of choice, a packing search to prune the results and *REFMAC* (Murshudov *et al.*, 2011 ▶) to perform an initial round of refinement] was then run using all of the chains of all the unique kinase structures in the PDB as search models (almost 2600 search models) and sampling all possible space groups in the Laue class. The output of the search revealed only one possible solution (Fig. 5 ▶): the sequence identity was less than 13%, but the C^α^ r.s.m.d. at the end was 1.18 Å, which is well below the theoretical 1.5 Å that indicates the limit for a successful search. An even more exhaustive way of taking advantage of the large and growing PDB is the use of *Wide Search Molecular Replacement* (Stokes-Rees & Sliz, 2010 ▶). Using this method, it was shown that by expanding the range of search models to the entire PDB, small (less than 12% structural coverage) and low sequence identity (less than 20% identity) templates can be identified through novel multidimensional template-scoring metrics and used to solve previously unknown macromolecular complexes. Although computationally very intensive, these workflows may become tractable through integration with national or international supercomputer grids. The potential lesson here is that whatever problem we are dealing with, the chances of finding a probe similar in three-dimensional structure, even if not necessarily in sequence, to our target protein are increasing, and model sampling and manipulation have become an essential part of the molecular-replacement procedure.

### 3.2. Other search models

Although the most commonly used search models are related X-ray structures, it should not be forgotten that other sources of structural information are available and that the advancements in several different techniques (from NMR to electron microscopy to *de novo* protein modeling) may make these models usable for MR.

#### 3.2.1. NMR

NMR is the second source of structural information for macromolecules: about 11% of the structures in the PDB have been solved using NMR, while X-ray structures represent about 88% of the entire content. The use of NMR models as probes for MR dates back to the mid-1980s (Brünger *et al.*, 1987 ▶); since then, there have been many successes but also several failures, mostly related to two major issues: the fact that the true solution may be buried among many incorrect solutions and thus difficult to extract, and failure during refinement (after a seemingly correct solution has been identified). Both issues are related to the differences between NMR and X-ray structures, mostly the fact that there could be genuine differences between a solution structure and that in a solid phase. For example, loops and termini are frequently not well defined, can assume different conformations or can be under-constrained owing to a lack of suitable NOE data; for the same reason, very elongated molecules can have long-range errors and not perform as well as search models as spherical structures. These problems can usually be addressed by the usual manipulation of the models such as the exclusion of disordered regions or side-chain truncation or by determination of the ‘best’ subset(s) of atoms. Another major problem could be the description of the correctness of the atomic positions, which in X-ray structures is expressed by temperature factors^3^ and is used during MR to properly weight individual atomic contributions to the scattering factors. A single NMR model lacks this information, but equivalent information is comprised in an NMR ensemble and can be incorporated either by building a single search model with artificial *B* factors derived from the atomic r.m.s.d. obtained when all structures in the ensemble are superimposed or by using the whole ensemble as a composite model (Chen *et al.*, 2000 ▶). Using the whole NMR ensemble seems to be a better approach, since it better describes the true time/space-averaged model (Müller *et al.*, 1995 ▶). In addition, in the past ten years or so there have been many improvements in the instruments and algorithms used for NMR structure determination, particularly in structural genomics projects, in which state-of-the-art software and assessment tools are routinely employed (Mao *et al.*, 2011 ▶). Combining NMR models and *Rosetta* refinement for model improvement can also significantly improve the phasing power of the models (Bin Qian *et al.*, 2007 ▶). Properly prepared NMR ensembles may show a performance very similar to X-ray structures when used as MR search models, particularly when the sequence identity is >40% (Mao *et al.*, 2011 ▶).

#### 3.2.2. Electron microscopy

X-ray diffraction represents the best method to obtain structural information at the atomic level, and in the most recent years, thanks to major improvements in crystallization, cryocooling, source intensity, data collection and processing, it has successfully been used to solve the structure of several large multiprotein complexes: in the PDB there are over 1100 structures with a molecular weight of greater than 500 000 Da that have been solved using X-­ray diffraction. Historically, these systems have mostly been analysed by single-particle cryo-electron microscopy (cryo-EM), in which a three-dimensional map at low resolution of the molecule is reconstructed by several two-dimensional projections (Baker *et al.*, 1999 ▶). Recent improvements in EM instruments and reconstruction techniques allow reconstruction at a resolution better than 10 Å to routinely be achieved and sometimes it is possible to approach near-atomic resolution (better than 3.5 Å; Zhang *et al.*, 2010 ▶, 2012 ▶; Ge & Zhou, 2011 ▶; Yu *et al.*, 2011 ▶). As a consequence, in the case of large complexes the two techniques can be combined (Dodson, 2001 ▶; Rossmann *et al.*, 2005 ▶), either by using an EM map to phase X-ray data or by combining EM data with information from X-ray crystallography or NMR spectroscopy to sort out the atomic details. In the first case, an EM image could be used as a search model in molecular-replacement processes. Initial low-resolution phases can then be extended to high resolution by density-modification techniques such as noncrystallographic symmetry (NCS) averaging. NCS occurs when macromolecules crystallize with more than one of the same molecule in the asymmetric unit of the crystal, which is fairly common when crystallizing large molecular complexes such as viruses, which are frequently highly symmetrical. MR with an EM map as a probe parallels the standard MR routine, the major difference being that the maps need to be scaled to the correct magnification, since the EM map is frequently deposited in the database on an arbitrary three-dimensional grid, resulting in an arbitrary size of the molecule. It can also be difficult to select the appropriate resolution range for both the EM and the X-ray data in the MR process: EM data are generally very good at low resolution (less than 25 Å) but fall off rapidly at higher resolution; X-ray data, on the other hand, rarely extend to very low resolution and also contain a significant scattering component from the solvent that should be accounted for (Navaza, 2008 ▶). Hence, the choice of the appropriate range for merging and scaling should be chosen carefully (Dodson, 2001 ▶). The review by Xiong (2008 ▶) reports a few test cases demonstrating how cryo-EM maps could be used as viable models for the MR solution of X-ray crystal structures. More details and examples on the use of low-resolution models to phase X-ray data can be found in Nicola Abrescia’s excellent contribution to the 2013 CCP4 Study Weekend (Stuart & Abrescia, 2013 ▶).

In the second case, atomic structures are docked into the electron-density map to yield a model of the complex. This has proven to be very useful for multimolecular structures such as complexes of ribosomes, tRNA and protein factors (Rawat *et al.*, 2006 ▶; Noeske & Cate, 2012 ▶) and muscle actomyosin (Rayment *et al.*, 1993 ▶). Fitting an atomic model into an EM map could be treated as regular molecular replacement, although there are some important differences that need to be considered (Navaza, 2008 ▶): (i) phase information is available, (ii) the symmetry of the EM reconstructions is not in general the point group of the crystallographic symmetry and (iii) the resolution of the EM images is usually low, which makes the identification of the molecular boundaries quite difficult. Nevertheless, several examples are available in which the combination of the two techniques has successfully provided a more detailed description of large macromolecular arrangements than either of the two techniques alone (Zhou, 2011 ▶; Allen & Stokes, 2013 ▶).

In order to facilitate the exchange of knowledge between X-­ray and electron microscopy, there has been an effort in both communities to make EM maps available in a standard format (the same CCP4 map format as used in X-ray crystallography); a public database, the Electron Microscopy Data Bank (EMDB), for three-dimensional EM data deposition (http://www.ebi.ac.uk/msd) has also been established and is directly linked to EM depositions in the Protein Data Bank.

#### 3.2.3. Small fragments and *de novo* models

One of the most common issues with molecular replacement is that often the search model does not have sufficient scattering power to generate a solution with a signal-to-noise ratio that is high enough to be identified. This may be owing to the fact that the search is run with one probe when there are multiple objects in the asymmetric unit or to the fact that the probe is a small fraction (a domain or less) of the actual final structure. How small can the search model be to still provide sufficient information during the search? It has been shown that even small portions of secondary-structure elements (α-helices, β-­strands and so on, accounting for less than 13% of the total structure), if correctly placed, can be used to successfully phase larger macromolecules (Yao *et al.*, 2005 ▶) when high-resolution data (better than 1.7 Å) are available. *ARCIMBOLDO* (Rodríguez *et al.*, 2009 ▶) has extended the concept to include structures with data up to 2 Å resolution. *ARCIMBOLDO* combines the location of small fragments (10–14-residue helices) with *Phaser* (McCoy *et al.*, 2007 ▶), density-modification expansion with *SHELXE* (Sheldrick, 2002 ▶) and autotracing of the resulting map with *REFMAC* (Murshudov *et al.*, 2011 ▶). The method is computationally expensive, but it can be parallelized to run on a grid or multiple processors (Rodríguez *et al.*, 2012 ▶). Five structures have been solved to date using *ARCIMBOLDO* (PDB entries 4aeq, 3gwh, 3szs, 4e1p and 3ufe; Usón *et al.*, 2012 ▶; Rodríguez *et al.*, 2009 ▶; Summers *et al.*, 2012 ▶; Peat *et al.*, 2012 ▶; C. Grosse, S. Himmel, S. Becker, G. M. Sheldrick & I. Usón, unpublished work); they are all mostly α-helical structures, but the method does not require the majority of the structure to be helical (Rodríguez *et al.*, 2009 ▶). The use of small fragments such helices as probes for MR can be successful even when only low-resolution (3.5 Å or less) data are available if there are other known restraints that can be used to prioritize solutions. For example, Strop *et al.* (2007 ▶) reported the successful phasing of the mechanosensitive channel of large conductance (MscL), a symmetric helical membrane protein, with only 4 Å resolution diffraction data. The probes used for MR were idealized transmembrane helices, and the large number of MR solutions was reduced by taking advantage of the known geometrical and structural restraints associated with membrane proteins.

If no experimental model is available, computational models of protein structures have proved to be useful as search models in MR (Raimondo *et al.*, 2007 ▶). It is obvious that the success of MR depends on the accuracy of the search model, and this is even more evident when protein models are used as probes. Giorgetti *et al.* (2005 ▶) reported that GDT_TS (the global distance test, which corresponds to the average value of the fraction of C^α^ atoms in the model that are placed within a distance of 1, 2, 4 or 8 Å from the corresponding C^α^ atoms in the native structure), was a better indicator of the model utility for MR than the r.m.s.d. They also showed that a GDT_TS of >84 was sufficient to guarantee MR success. This parameter gives a measure of the global (overall) accuracy of the model, but unfortunately it is unknown until the structure of the target has been solved. To overcome this issue, in the structural bioinformatics community, model-quality assessment programs (MQAPs) have been developed which predict the accuracy of protein structure models without knowledge of the true structure, both globally and for individual residues. MQAPs are based either on physical effective energy obtained from fundamental analysis of the particle forces or on empirical pseudo-energy derived from known protein structures (Lazaridis & Karplus, 2000 ▶). In addition, MQAPs can be divided into ‘true MQAPs’, which are methods that are capable of assessing quality for single models without using any alternative models (decoys) for the target protein, and ‘clustering MQAPs’, which are methods that rely on structural comparisons between a number of alternative models generated for the target sequence. When plenty of models are available the clustering approaches significantly outperform the true MQAPs, especially when ranking models according to their accuracy, but the gap between the two methods is only marginal when only one or only a few models are available (McGuffin, 2007 ▶; Pawlowski *et al.*, 2008 ▶). Comparative models perform much better in MR if they are used together with the predicted local accuracy, and the lower the global quality of a model is the more significant is the impact of knowledge about the local quality (Pawlowski & Bujnicki, 2012 ▶).

## 4. Conclusions

Molecular replacement has come a long way from the initial programs in accuracy, speed, ease of use and level of automation. Thanks to advances in computing power and software developments, molecular replacement has become possibly the most user-friendly technique available. While major efforts are still under way in software improvement, more and more work is focusing on the preparation of search models. There is clearly a direct relationship between the accuracy and quality of the chosen probe and its usefulness as a search model. Accuracy is probably the most difficult component to assess: sequence identity and model-quality assessment programs can be useful to evaluate the accuracy of the probes but will not give a definitive answer, which is dependent on the solution itself. On the other hand, the quality of the probes is something that can easily be measured and controlled. The introduction of cross-validation by *R*_free_ (Brünger, 1992*a* ▶) had a large impact in validating the structures deposited in the Protein Data Bank as well as providing a tool to discriminate correct from incorrect solutions, even with difficult systems (Strop *et al.*, 2007 ▶) and at very low resolution (Brunger *et al.*, 2012 ▶). Numerous other tools for assessing the quality of structures are available; for example, *MolProbity* (Chen *et al.*, 2010 ▶) and the *WHAT IF* server (Vriend, 1990 ▶; Hekkelman *et al.*, 2010 ▶). Laskowski (2003 ▶) provided an exceptionally clear and succinct overview of how to evaluate model quality, and other reviews are available (for example, Kleywegt, 2007 ▶). A concerted effort should be made by the entire structural biology community to ensure that only the best possible models are deposited in the databases, since minor differences in the quality of the model can have substantial effects on the final outcome.

## Footnotes

1 Throughout the paper, the term ‘quality of the model’ refers to how good the structure is, and this is usually judged by the resolution, the *R*/*R*_free_ values of the final model and all of the other useful statistics (r.m.s.d. bonds and angles, Ramachandran plot, geometry, clash scores *etc.*) associated with a structure. These parameters have been consistently improving over the years: for example, over 67% of all structures at 2.5 Å resolution or better available in the PDB have been deposited since 2005, about 31% were deposited between 2000 and 2005, and only 2% were deposited before 2000. Of these structures, about 16% report an *R*_free_ better than 20%; of these, 81% have been deposited after 2005.

2 Molecular replacement is basically a six-dimensional problem: the coordinates of the structure to be solved are the transformation of the coordinates of the search model to which a rotation (defined by three angles) and a translation (define by the three vectors) are applied. Because of the size of the models and the size of the space to be searched, performing a six-dimensional search is a very time-consuming process. For example, for a typical unit cell (orthorhombic, 100 × 100 × 100 Å) and a fairly coarse grid (5° rotation increments, 1 Å translation increments) a six-dimensional search will have 36 × 36 × 18 × 50 × 50 × 50 = 3 × 10^9^ points. Even if each point took 0.01 s to evaluate, that would still require 8100 h of computer time.

3 The temperature factor is a factor that describes the degree to which the electron density is spread out around atom *i*, and it is proportional to the mean-square displacement of the atom *i* (*U*). As *U* increases, *B* increases and the contribution of the atom to the scattering is decreased. Incorrectly built atoms will tend to have higher *B* factors than correctly built atoms nearby.
